# Quorum Sensing Negatively Regulates Multinucleate Cell Formation during Intracellular Growth of *Burkholderia pseudomallei* in Macrophage-Like Cells

**DOI:** 10.1371/journal.pone.0063394

**Published:** 2013-05-21

**Authors:** Rachel E. Horton, Gary D. Grant, Ben Matthews, Michael Batzloff, Suzzanne J. Owen, Stephanie Kyan, Cameron P. Flegg, Amanda M. Clark, Glen C. Ulett, Nigel Morrison, Ian R. Peak, Ifor R. Beacham

**Affiliations:** 1 Institute for Glycomics, Griffith University, Gold Coast, Queensland, Australia; 2 School of Pharmacy, Griffith University, Gold Coast, Queensland, Australia; 3 Smart Water Research Centre, Griffith University, Gold Coast, Queensland, Australia; 4 School of Medical Science, Griffith University, Gold Coast, Queensland, Australia; East Carolina University School of Medicine, United States of America

## Abstract

*Burkholderia pseudomallei* is a Gram-negative environmental bacterium and the causative agent of melioidosis, a potentially fatal, acute or chronic disease endemic in the tropics. Acyl homoserine lactone (AHL)-mediated quorum sensing and signalling have been associated with virulence and biofilm formation in numerous bacterial pathogens. In the canonical acyl-homoserine lactone signalling paradigm, AHLs are detected by a response regulator. *B. pseudomallei* encodes three AHL synthases, encoded by *bpsI*1, *bpsI*2 and *bpsI*3, and five regulator genes. In this study, we mutated the *B. pseudomallei* AHL synthases individually and in double and triple combination. Five AHLs were detected and quantified by tandem liquid chromatography-mass spectroscopy. The major AHLs produced were *N*-octanoylhomoserine lactone and *N*-(3-hydroxy-decanoyl)homoserine lactone, the expression of which depended on *bpsI*1 and *bpsI*2, respectively. *B. pseudomallei* infection of macrophage cells causes cell fusion, leading to multinucleated cells (3 or more nuclei per cell). A triple mutant defective in production of all three AHL synthases was associated with a striking phenotype of massively enhanced host cellular fusion in macrophages. However, neither abrogation of host cell fusion, achieved by mutation of *bimA* or *hcp*1, nor enhancement of fusion altered intracellular replication of *B. pseudomallei*. Furthermore, when tested in murine models of acute melioidosis the AHL synthase mutants were not attenuated for virulence. Collectively, this study identifies important new aspects of the genetic basis of AHL synthesis in *B. pseudomallei* and the roles of these AHLs in systemic infection and in cell fusion in macrophages for this important human pathogen.

## Introduction

Melioidosis is a potentially fatal disease endemic to the tropics, particularly North Eastern Thailand and Northern Australia, where the mortality rates approach 14% in Australia and 50% in Thailand [1]–[3]. The incidence of disease in the Northern Territory of Australia was 15.5 per 100,000 between 2001 and 2002 and 2.7 times that rate amongst indigenous Australians [2], [3]. Melioidosis is also considered to be under-reported and an emerging disease worldwide, with infections reported in Brazil, China, India and Malaysia [4]–[9]. Clinical presentations of melioidosis vary, ranging from chronic to acute disease and potentially rapid death due to systemic infection and septic shock [1]. Virtually any organ of the body may be infected; while skin and soft tissues including lungs, liver and spleen are often involved, the parotid gland, the central nervous system and bone may also be infected [1], [3].

*Burkholderia pseudomallei*, the causative agent of melioidosis, is easily isolated from soil and standing water in regions of endemicity. Due to potential infection *via* aerosolisation [1], [10], the current lack of a vaccine, a capacity for relapse and latent infection [11], intrinsic antibiotic resistance, and its ease of isolation and culture from the environment, *B. pseudomallei* is listed as a category B potential biowarfare agent [12].

Infection of humans stems from environmental exposure and is closely associated with high rainfall [10]. Two common routes of inoculation are thought to be percutaneous and inhalation. Aside from the hazards of working in standing water, such as rice paddies, inhalation of contaminated water droplets or dust (soil) is probably the most important natural route of infection. Animal studies of melioidosis using inhalational exposure and intranasal inoculation demonstrate infection of the lung and systemic infection of internal organs. Such studies have revealed previously unrecognized sites of colonization in the nasal associated lymphoid tissue (NALT) and in the nasal mucosa, especially the olfactory epithelium (OE). This suggests that respiratory infection and nasal colonization may be a portal of entry to the brain and blood without necessarily involvement of lower respiratory tract [13].

Several virulence factors of *B. pseudomallei* have been well defined in mice and hamsters, and in *in vitro* models of infection. These include the capsule, lipopolysaccharide, and Type III and VI secretion systems [14]–[16]. *In vitro* models of infection include murine and human phagocytic cell lines in which *B. pseudomallei* efficiently replicates [14]. *B. pseudomallei* is phagocytosed and replicates in RAW264.7 cells, a murine macrophage-like cell line in which *B. pseudomallei* is also noted for its capacity to induce cell fusion, leading to multinucleate cell (MNC) formation [17]–[19]. MNC formation is also observed in lung tissue in a murine model of chronic human melioidosis [20]. Both MNC formation and intercellular spread involve actin-mediated motility in epithelial cell lines [14], [15], [21]. Such motility requires the *bimA gene*, which encodes a polar membrane protein required for actin polymerisation [18], and one of six Type VI secretion systems (encoded by *hcp1* within T6SS-1) is required for cell fusion and MNC formation [15], [22]. However, the exact role of MNC formation in pathogenesis is not established [20].

One of the most well studied intercellular bacterial communication systems is based on acyl-homoserine lactone (AHL)-mediated quorum sensing (QS). This is widespread in Gram-negative bacteria and is associated with pathogenicity in organisms such as *Pseudomonas aeruginosa* [23], [24]. In this system, AHLs diffuse from the cells and accumulate in a cell-density-dependent manner in the growth medium (or environment); diffusion back into the bacteria from the external milieu results in binding to a cognate regulator protein and coordinated regulation of genes in the bacterial community. In *B. pseudomallei*, there are three AHL synthases, encoded by the bpsI genes, and a number of regulatory genes such as *bps*R [25]. Analysis of mutants lacking these genes has implicated AHL-mediated signaling in the regulation of extracellular enzyme and siderophore formation, the oxidative stress response, and biofilm formation [26]–[29]. In addition, a bpsI mutant was attenuated in a *Caenorhabditis elegans* model [27], and in hamsters and mice [25], [26].

In this study, we consider the hypothesis that QS plays a role in *B. pseudomallei* virulence *in vitro*, in relation to MNC formation, and in vivo, situations in which high bacterial cell densities are achieved. We focused on *bpsI* mutants to determine the potential roles of QS in pathogenicity, and a triple *bpsI* mutant in which no AHL-mediated QS is possible. For our *in vivo* studies we used upper respiratory tract (URT), lung, and brain infection models. The results show that whilst there is no significant role of QS for *in vivo* virulence in these models, there is a major effect on MNC formation in macrophage-like cells.

## Materials and Methods

### Bacterial Strains, Culture Conditions and Antibiotics

The bacterial strains used in this study are described in Table 1. Bacteria were grown at 37°C on lysogeny broth (LB) agar or in LB broth in shaken culture. Antibiotics, when appropriate, were added at the following concentrations: ampicillin, 100 µg ml^−1^, chloramphenicol, 50 µg ml^−1^ (*E. coli*) or 100 µg ml^−1^ (*B. pseudomallei*), gentamicin, 15 µg ml^−1^, streptomycin, 100 µg ml^−1^, tetracycline, 10 µg ml^−1^, and trimethoprim 100 µg ml^−1^.

**Table 1:** Bacterial strains used in the study.

Species/strain	Genotype/phenotype	Reference
*Burkholderia pseudomallei*		
MSHR520	Wild-type; clinical isolate	Owen et al., 2009
MSHR520 Δ*cap*	Δ*cap*; Tc^r^	Owen et al., 2009
MSHR520 Δ*bpsI*1	Δ*bpsI1*	This study
MSHR520 Δ*bpsI*2	Δ*bpsI2*	This study
MSHR520 Δ*bpsI*3	Δ*bpsI3*	This study
MSHR520 Δ*bpsI*12	Δ*bpsI12*	This study
MSHR520 Δ*bpsI*13	Δ*bpsI13*	This study
MSHR520 Δ*bpsI*23	Δ*bpsI23*	This study
MSHR520 Δ*bpsI*123	Δ*bpsI123*	This study
MSHR520 Δ*cap* Δ*bpsI*1	Δ*bpsI1* Δcap; Tc^r^	This study
MSHR520 Δ*cap* Δ*bpsI*2	Δ*bpsI2* Δcap; Tc^r^	This study
MSHR520 Δ*cap* Δ*bpsI*3	Δ*bpsI3* Δcap; Tc^r^	This study
MSHR520 Δ*cap* Δ*bpsI*123	Δ*bpsI123* Δcap; Tc^r^	This study
MSHR520 Δ*cap bimA*	Δ*bimA* Δ*cap*; Tc^r^	This study
MSHR520 Δ*cap hcp1*	Δ*hcp1* Δ*cap*; Tc^r^	This study
*Escherichia coli*		
DH5α	*SupE*44 Δ*lacU*169 (Ф80 *lacZ*ΔM15) *hsdR*17 *recA*1 *endA*1 *gyrA*96 *thi*-1 *relA*1	
S17.1(λpir)	RP4-2-Tc::Mu Km::Tn7 Tp Sm (λpir) *pho*A20	

### Mutagenesis

Unmarked in-frame deletion mutagenesis was used to create mutants by allelic exchange [30]. Mutant alleles with a deletion within the relevant gene were synthesised and cloned in pUC57 (Genscript, USA). Deletions encompassed the following nucleotide positions, with reference to the genome sequence of K96243 [31]: *bimA* (BPSS1492, 2032913–2036300; deletion between 2033879 and 2035245), *hcp* (BPSS1498, 2042122–2044528; deletion between 2043093 and 2043496), *bpsI1* (BPSS0855, 1176590–1179016; deletion between 1177565 and 1178064), *bpsI2* (BPSS1180, 1587232–1589606; deletion between 1588246 and 1588739), and *bpsI3* (BPSS1570, 2130500–2132616; deletion between 2131330 and 2131811). Mutant alleles were subcloned into pDM4-Tp [30] and then conjugatively mobilised from *Escherichia coli* S17.1 (λ*pir*) into *B. pseudomallei* MSHR520, obtained from Bart Currie, Menzies School of Health Research, Darwin, Australia (previously referred to as strain 08 [19], [32]). Single cross-over integrants were selected on chloramphenicol and screened for trimethoprim resistance. Clones in which the integrated deletion construct was excised by a second recombination event was then selected as sucrose-resistant. This was confirmed by chloramphenicol and trimethoprim sensitivity and clones with the deleted allele determined by PCR analysis with appropriate primers. Double and triple mutants are denoted, for example, as *BpsI*12 (mutated in *bpsI*1 and *bpsI*2), *bpsI*123 (mutated in all three *bpsI* loci) etc.

### Analysis of AHL Production

Bacteria were grown to late log-early stationary phase (A_600_ 1.2) at 37°C at 180 rpm. Cultures were then centrifuged and the supernatant filtered through a 0.22 µm PVDF filter (Millipore). *N*-septanoyl-L-homoserine lactone (C7-HL; 100 ng ml^−1^) was added, to allow correction for extraction efficiency, prior to extraction twice with equal volumes of ethyl acetate acidified with 0.1% glacial acetic acid. Aspirated ethyl acetate was evaporated under nitrogen at 35°C and reconstituted in LCMS grade methanol containing *N*-nonanoyl-L-homoserine lactone (C9-HL; 1 µg ml^−1^) as internal standard. Chromatographic separation was performed using 2 µl injections onto an Agilent HPLC fitted with a Phenomenex Kinetics XB-C18 column (2.1 mm×100 mm with a 1.7 µm particle size) prior to analysis on an Agilent 6530 Q-TOF using a dual injection Jet Stream ESI source.

The chromatographic conditions consisted of the following mobile phases at a flow rate of 0.5 ml/min A: ultrapure water, and B: LCMS grade acetonitrile (Fisher), both buffered with 0.1% glacial acetic acid. The chromatographic cycle consisted of a linear gradient from 20% B to 90% B over 10 min followed by isocratic column flush at 90% B for 2 min and column re-equilibration at 20% B for a further 1 min. Mass spectroscopy (MS) analysis was performed in positive ion mode. Nitrogen was used as a drying and sheath gas, which was heated to 250°C and 325°C with flow rates of 12 l/min and 10 l/min, respectively. The capillary and fragmentor were set to 2500 V and 135 V. MS data was acquired in wide dynamic range mode between 100 and 1700 *m/z* with an acquisition rate of 1.4 spectrums sec^−1^ examining 9665 transients per spectrum. Quantitation of AHLs was performed in MS only mode against extracted ion chromatographs at 5 ppm mass accuracy with a minimum of 7 acquisitions per peak using the Agilent Mass Hunter Quantification Analysis software package. Quantification of all AHLs was performed using the ratio of peak area of analyte to that of the C9 internal standard and compared to 10 point standard curves.

Validation of analytes was done using a modified method of that reported by Gould *et al.* [33]. In brief, MS/MS fragmentation data was acquired at 1.4 spectrums sec^−1^ with 9665 transients per spectrum. The collision induced disassociation energy for fragmentation was determined according to *m/z* of the ion being analysed using the slope determination function of the Mass Hunter acquisition software using a slope of 3.7 and an offset of 9. MS/MS data analysis was performed using Agilent Qualitative analysis software. Positive confirmation occurred if the precursor ions examined displayed mass accuracies of 5 ppm or less to the analyte of interest and the 102.0550 *m/z* fragment ion corresponding to the lactone ring structure common to the AHLs was present.

### *In vitro* Infection of Macrophage-like Cells

RAW264.7 cells (ATCC TIB-71) were grown in DMEM (Gibco), supplemented with 10% heat-inactivated FBS (Gibco) at 37°C with 5% CO_2_. Cells were seeded at 5×10^4^ cells per 2.0 cm^2^ growth area into 96 or 24 well cell culture plates 24 h prior to infection. Cells were infected with bacteria grown for 16 h at a multiplicity of infection of 10 bacteria per cell and incubated at 37°C with 5% CO_2_ for 1 h. Culture medium was removed and replaced with medium containing 10 µg ml^−1^ imipenem (Sigma) to suppress extracellular bacterial replication [19], [34]. Incubation was continued for up to 24 h. To quantify intracellular survival and replication infected RAW264.7 cells in 96 well tissue culture plates were washed with phosphate buffered saline (PBS) and permeabilised using 0.1% triton X-100. Released cell–associated bacteria were enumerated by plating and colony counts after 18–20 h growth.

### Assessment of Multinucleated Cell Formation

Infected RAW264.7 cells in 24 well tissue culture plates were washed with PBS and immediately fixed with 3.7% (v/v) formalin and stored at 4°C with added PBS until use. For enumeration of nuclei and multinucleation, cells were stained in the wells using 10% Giemsa for 10 min, washed with PBS and air-dried before viewing under 200× magnification. Ten fields of view or at least 1000 nuclei were counted for each well. Both numbers and sizes of MNCs, defined as containing 3 or more nuclei per cell, were recorded. For immunofluorescence, the monolayers were grown and infected on glass coverslips within 24 well plates, washed 3 times with PBS and permeabilised for 10 min with 0.1% triton X-100. All subsequent stages took place at room temperature and were each followed by 3 washes with PBS. Blocking was 30 min with 5% (w/v) BSA in PBS, followed by incubation with a polyclonal rabbit *B. pseudomallei* antisera [19] for 30 min, followed by incubation with phalloidin-alexa 568 conjugate (Invitrogen) and an anti-rabbit IgG Alexa 488 conjugate (Invitrogen) for 30 min and then 10 min incubation with DAPI (Invitrogen). Coverslips were dried and mounted onto slides using n-propyl gallate (Sigma). Slides were stored at 4°C until viewing.

### Animal Studies

BALB/c 5 week old mice (n = 9) were infected intranasally with 3×10^5^ cfu for each strain of *B. pseudomallei*, with inoculating doses confirmed by serial dilutions and enumeration on agar. For investigation of URT, mice were euthanised at the stated time intervals post-infection by lethabarb (pentobarbital sodium) injection, and blood, NALT, olfactory bulbs (OB) and OE were collected for enumeration of *B. pseudomallei*; for investigation of systemic infection, blood, lungs, liver and spleen were harvested [13]. Ethical approval for animal experimentation was granted by the Griffith University Animal Ethics Committee, in accordance with National Health and Medical Research Council (NHMRC) of Australia guidelines.

### Statistics

Data were compared between groups using a non-parametric ANOVA (Kruskal-Wallis test) using a Dunns post test with significance set at p<0.05. Analyses were carried out using Prism version 5.0 (GraphPad Software).

## Results

### *B. pseudomallei*-triggered MNC Formation is Abrogated by Deletion of *bimA* or *hcp1* and is Independent of Intracellular Replication

**Figure 1:**
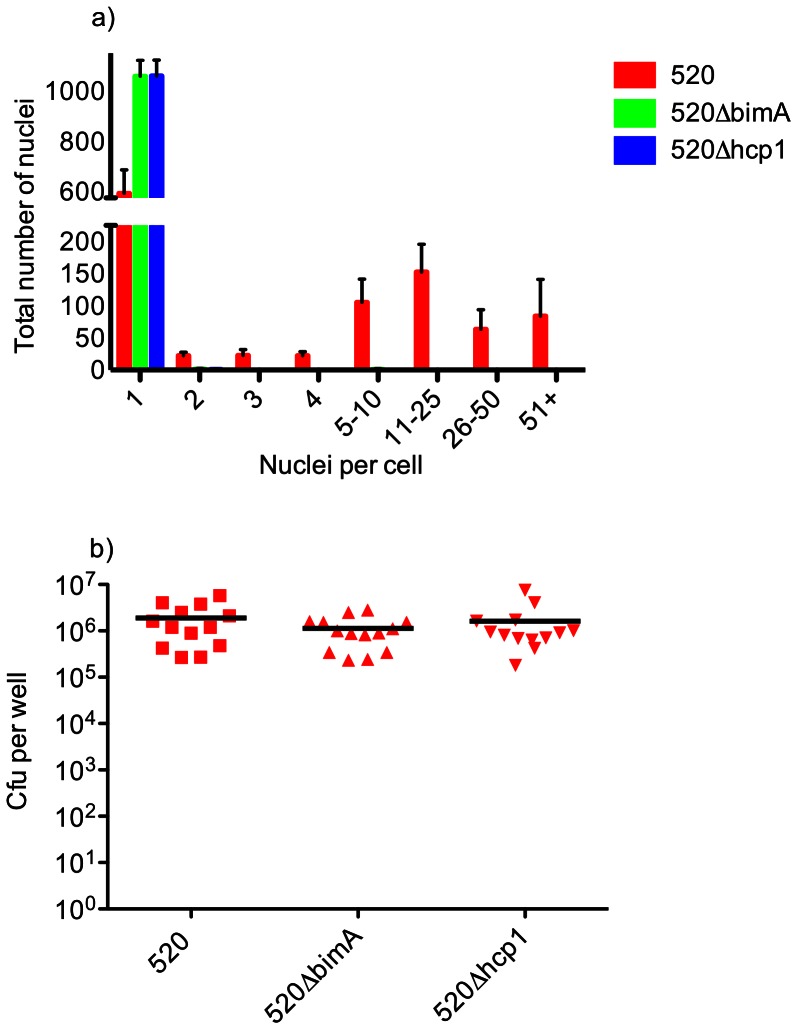
Infection of RAW264.7 cells by *B. pseudomallei* MSHR520, MSHR520Δ*bimA*, and MSHR520Δ*hcp*1. Infections were carried out over a period of 12 h and fixed for Giemsa staining (a) or lysed to release internalised bacteria, which were then plated for enumeration of cfu per well (b). (a) Formation of MNCs in cells infected with Δ*bimA* and Δ*hcp*1 mutants as compared with wt (MSHR520). Comparisons between mutants and wt were statistically significant (P<0.001). >1000 nuclei per well were counted and are expressed as number of nuclei in each category. Columns show the mean values with SEM. (b) Intracellular bacteria in cells infected with wt, Δ*bimA* and Δ*hcp*1 mutants as compared with wt (MSHR520). No significant difference (P = 0.46) is observed between wt and Δ*bimA* or wt and Δ*hcp*1. Scatter plots show each well as an individual point with the mean value represented by the horizontal bar. Data are derived from 4 separate experiments, each including duplicate or triplicate wells.

**Figure 2:**
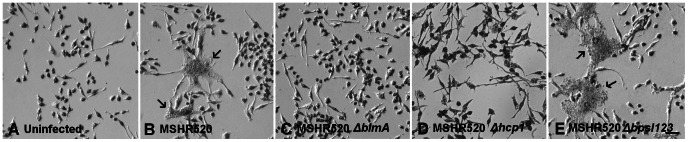
Representative views of *B. pseudomallei* infected RAW264.7 cells at 12 h post infection. A) Uninfected; B) MSHR520 (wt) infected; C) MSHR520 Δ*bimA*; D) MSHR520 Δ*hcp*1; E) MSHR520 Δ*bpsI*123. Cells were fixed with 3.7% (w/v) PFA and stained with Giemsa. Arrows in B and E indicate MNCs. Scale bar in E represents 50 µm.

**Figure 3:**
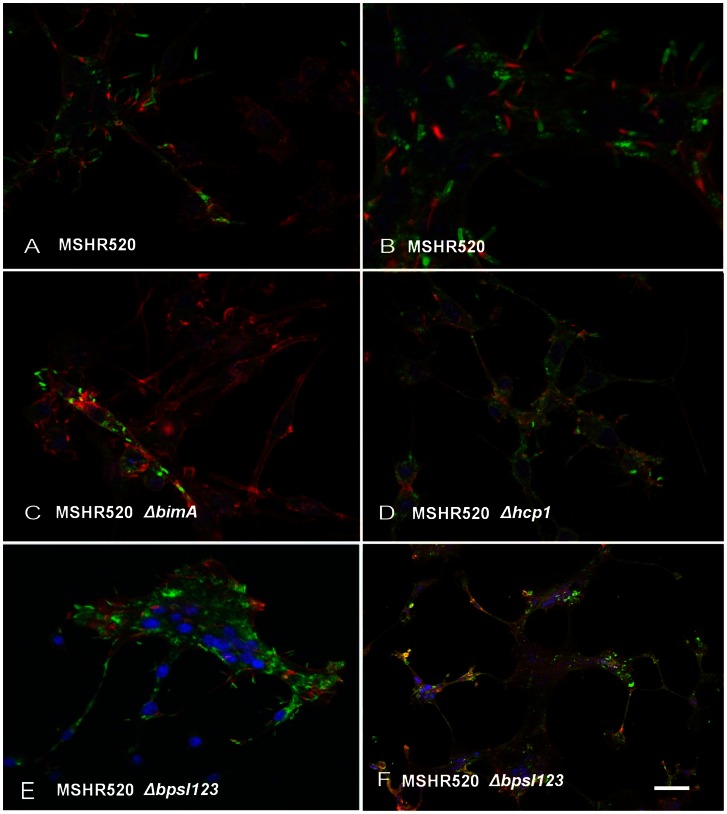
Representative views of *B. pseudomallei* infected RAW264.7 cells at 12 h post infection. A) & B) MSHR520 (wt) infected; C) MSHR520 Δ*bimA*; D) MSHR520 Δ*hcp*1; E) & F) MSHR520 Δ*bpsI*123. Nuclei are stained blue with DAPI; bacteria are labelled green with rabbit anti-*B. pseudomallei* IgG and anti-rabbit IgG-alexa 568; and actin is labelled red with phalloidin-alexa 488. Red actin tails, at the poles of bacteria, are visible in all panels with the exception of C. Scale bar represents 25 µm (A, C, D, E), 12.5 µm (B), 250 µm (F).

In the course of utilising RAW264.7 cells to investigate the role of QS in intracellular replication, we examined whether cell fusion is required for optimal intracellular replication. We first confirmed that a mutant lacking Hcp1 [22], encoded within the T6SS-1 locus, failed to induce significant MNC formation in infected RAW264.7 cells up to 12 h post-infection (Figure 1a; Figures 2, panel D, & Figure 3 panel D). Similarly, almost complete abrogation of MNC formation was seen when actin-based motility was prevented by deletion of *bimA* (Figure 1a; Figures 2, panel C, & Figure 3 panel C). However, no significant differences in numbers of intracellular bacteria were seen between the wild-type (parental; wt) and mutant strains (Figure 1b), indicating that intracellular replication is independent of MNC formation.

### Effect of Deletion of *bpsI1*, *2* & *3* on AHL Production

Homologues of LuxI proteins are responsible for the biosynthesis of AHLs that mediate bacterial QS. In-frame deletion mutants of all three *B. pseudomallei bpsI* homologues (BPSS0855, BPSS1180, BPSS1570) were constructed singly and in combination, and the AHLs produced by these strains assessed by LCMS/MS analysis of culture supernatant (Table 2). *N*-octanoylhomoserine lactone (C8HL) and *N*-(3-hydroxy-decanoyl)homoserine lactone (OHC10HL) were the predominant AHLs found, constituting nearly 90% of the total AHLs in the wt strain. The AHLs, *N*-(3-hydroxy-octanoyl)homoserine lactone (OHC8HL), N-decanoylhomoserinelactone (C10HL) and *N*-(3-hydroxy-dodecanoyl)homoserine lactone (OHC12HL) were also present in significant amounts. C8HL was absent (<2%) in the *bpsI*1 mutant, and all other *bpsI*1-deficient strains (*bpsI*12, *bpsI*13, *bpsI*123). Likewise, C10HL, OHC10HL and OHC12HL were absent in the single *bpsI*2 mutant and all other *bpsI*2 mutant strains. The production of OHC8HL by the mutant strains was more complex: All three single mutants and *bpsI*31 expressed less OHC8HL but this AHL was not detected in cultures of *bpsI*12, *bpsI*32 and *bpsI*123; together, these data show a role for *bpsI*2 in OHC8HL synthesis, but in conjunction with *bpsI*1 and/or *bpsI*3.

**Table 2:** AHLs produced by *B. pseudomallei* MSHR520 and its derived mutants.

AHL	Extracellular AHLs detected (nM)							
	MSHR520	*bpsI*1	*bpsI*2	*bpsI*3	*bpsI*12	*bpsI*13	*bpsI*23	*bpsI*123
C8HSL	8647±1222	129±191	5178±2496	7002±4841	ND	ND	12110±4218	ND
OHC8HSL	1350±587	778±224	486±227	455±307	ND	650±45	ND	ND
C10HSL	623±142	335±178	ND	469±260	ND	390±70	ND	ND
OHC10HSL	10966±4233	8583±3697	ND	6421±5164	ND	9561±1520	ND	ND
C12HSL	ND	ND	ND	ND	ND	ND	ND	ND
OHC12HSL	284±229	399±175	ND	ND	ND	449±177	ND	ND

Mutant strains are deleted in each AHL synthase gene indicated, either singly or in double or triple combination. Each value is presented as the mean ± SD. ND, not detected.

### AHL Synthase-deficient *B. pseudomallei* Induces Mass MNC Formation in Macrophages

**Figure 4:**
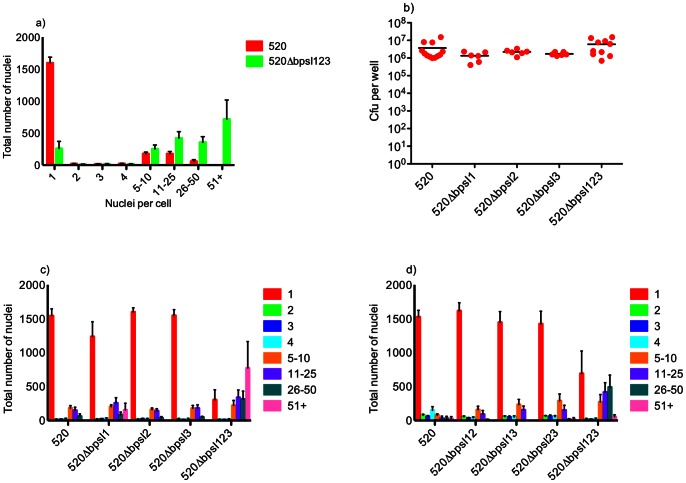
Infection of RAW264.7 cells by *B. pseudomallei* MSHR520 and related mutants. Infections were carried out over a period of 12 h and fixed for Giemsa staining (a), (c), (d), or lysed to release internalised bacteria which were then plated for enumeration of cfu per well (b). (a) Formation of MNCs is increased in the triple Δ*bpsI*123 mutant as compared with wt (MSHR520); comparison of mutant to wt is statistically significant for the 51+ group. >1000 nuclei were counted for each well and are expressed as the number of nuclei per group. Columns show the mean values with SEM. (b) No significant variation in intracellular bacteria is seen between wt and Δ*bpsI*1, Δ*bpsI*2, Δ*bpsI*3, Δ*bpsI*123. (c) MNC formation in single AHL mutants: In contrast to the triple mutant strain (comparison of mutant to wt is statistically significant for the 51+ group), formation of MNCs does not significantly differ between the wt and any of the single AHL mutants. (d) MNC formation in double AHL mutants: In contrast to the triple mutant strain (comparison of mutant to wt is statistically significant for the 11–25 and 26–50 groups), formation of MNCs does not differ between the wt and any of the double AHL mutants. Data in each figure are derived from 3 separate experiments, each including duplicate or triplicate wells.

Since cultured cells may involve cell density-mediated QS, we next examined the effect of abrogation of AHL synthesis on the capacity of RAW264.7 cells to support replication of *B. pseudomallei*, and undergo cell fusion leading to MNC formation. We quantified MNC formation by enumerating the numbers of cells with multiple nuclei, in cultures infected with the wt strain or mutants with *bpsI* deleted individually, in pairs, or mutation in all three *bpsI* loci. We observed a remarkable increase in cellular fusion induced by the triple *bpsI* mutant, (Figure 4 a, c, d; and see Figures 2, panel E, and 3 panels E & F). This was manifested as a marked and significant increase in the number of nuclei associated with cells containing >10 nuclei, as compared to the wt. Experiments using the mutants lacking only one or two AHL synthases failed to show any significant differences compared to the wt strain in terms of MNC formation (Figure 4 c, d). Interestingly, enumeration of intracellular bacteria showed no significant differences in intracellular survival/replication between the wt strain and mutants lacking AHL synthases, including the triple *bpsI* mutant (Figure 4b).

Representative images of RAW264.7 cells infected with MSHR520 and isogenic mutants are shown in Figures 2 and 3, either Geimsa stained or subject to IHC, respectively. Typical MSHR520-induced MNCs are shown with actin tail formation (Figure 2 panel B; Figure 3 panels A & B); MNCs were not evident following infection with *bimA* or *hcp1* mutants (nor were actin tails observed in the former case), although infected cells were observed (Figures 2 & 3 panels C & D). Very large MNCs (>10–50 nuclei), induced following MSHR520 infection were substantially more numerous in *bpsI*123-infected cells (Figure 4), and are shown in Figures 2 (panel E) and 3 (panels E & F).

### AHL Synthase Mutants Retain Virulence Following Intranasal Infection in Mice

**Figure 5:**
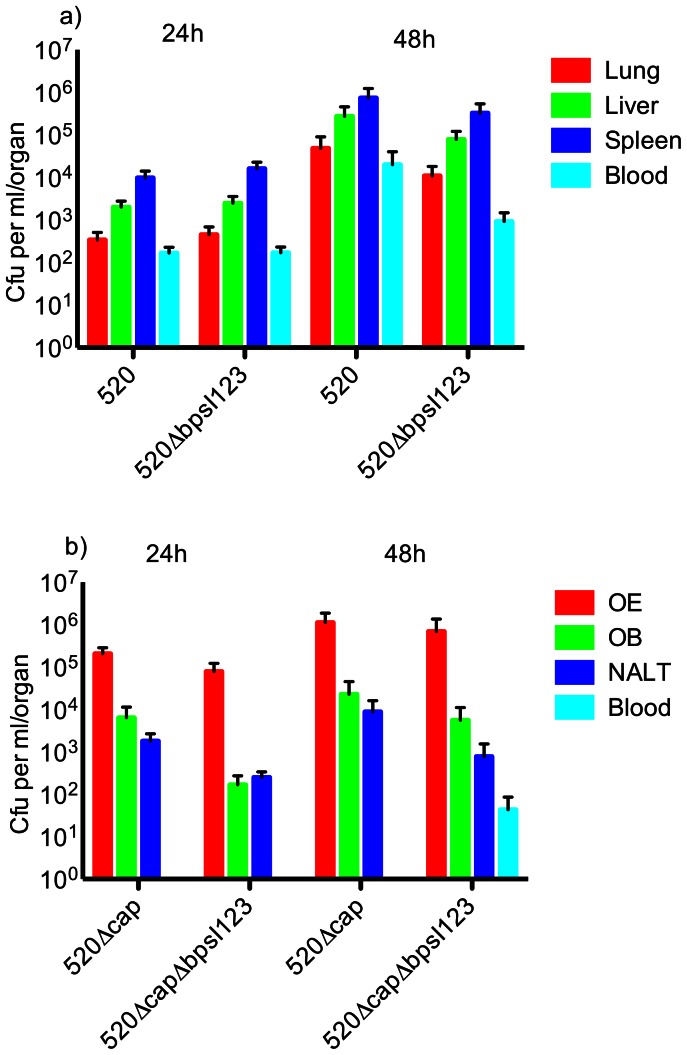
Infection of BALB/c mice with *B. pseudomallei* MSHR520 and related mutants. (a) Infection with MSHR520 and MSHR520Δ*bpsI*123 followed by determination of bacterial loads in systemic sites. (b) Infection with MSHR520Δcap and MSHR520Δcap Δ*bpsI*123 followed by determination bacterial loads in the upper respiratory tract (URT), olfactory epithelium (OE), olfactory bulb (OB), nasal-associated lymphoid tissue (NALT), and blood. No significant differences were observed between wt and mutant strains for equivalent tissues and time points.

To assess whether abolishing AHL synthesis might affect pathogenicity in an acute infection model, BALB/c mice were challenged intranasally and organ loads were enumerated post-infection. No significant differences were observed in bacterial numbers between mice infected with the wt strain, and the *bpsI*123 mutant in lung, liver, spleen, or blood (Figure 5a).

Finally, to determine if infection of the URT, and subsequent infection of the OB, might be affected by disrupting QS signalling in *B. pseudomallei*, infections with acapsulate AHL synthase mutants were assessed. In these assays, the confounding factor of haematogenous spread to the brain was reduced, since acapsulate strains have a lower capacity to survive in the blood, as we previously demonstrated [13]. However, as observed with capsulate strains, the capacity of AHL synthase mutants to colonise and survive in the URT, and the OB, was not significantly different compared to the wt strain (Figure 5b).

## Discussion

This study establishes that C8HL and OHC10HL are the predominant AHLs produced by *B. pseudomallei* MSHR520, constituting 90% of the total detected AHLs, and are produced in approximately equal concentrations in aerated broth cultures. The AHLs OHC8HL, C10HL and OHC12HL are also present in significant amounts. A difference in our findings compared to a prior study [29] is that we observed distinct relative amounts of C8HL and OHC10HL. Since C8HL is virtually absent in the *bpsI*1 mutant, and not detected in all other *bpsI*1-deficient strains (*bpsI*12, *bpsI*13, and *bpsI*123; Table 2), we conclude that BpsI1 is responsible for synthesis of C8HL. Likewise, we conclude that BpsI2 is responsible for the synthesis of C10HL, OHC10HL and OHC12HL since they are absent in the single *bpsI*2 mutant and all other *bpsI*2 mutant strains. The pattern for OHC8HL is more complex, with reduced production but not absence in each of the single *bpsI* mutants and in *bpsI*31. OHC8HL was not detected in cultures of *bpsI*12, *bpsI*32 and *bpsI*123, indicating that *bpsI*2 is required but that *bpsI*1 and/or *bpsI*3 are also required for full production. It was reported that transcription of *bpsI*3 depends on *bpsI*1 in stationary phase [29] and thus it may be that OHC8HL depends on both *bpsI*2 and *bpsI*3. Our results are consistent with prior data derived using single mutants, although the AHL levels of two of the single mutants were undetected rather than reduced [29]. Dependence on two synthases might be via an unknown mechanism, or via both acting independently in the synthesis of OHC8HL.

*B. pseudomallei*, *B. thailandensis* and *B. mallei* can invade and replicate inside mammalian cells, and induce cellular fusion. A striking and novel finding in the current work was that complete abolition of AHL synthesis in the triple mutant (*bpsI*123) resulted in a remarkable enhancement of cellular fusion in RAW264.7 cells. By comparing the AHL mutants with the wt strain, and two mutants (*bimA* and *hcp1*) previously reported to be unable to induce cellular fusion in epithelial cell lines [15], [18], [21], [22] we showed this phenotype to be independent of intracellular bacterial survival and replication. Despite inducing increased MNC formation, the AHL synthase mutants were not more proficient in intracellular replication. The *hcp1* and *bimA* mutants, which did not induce MNCs, were also unaffected in intracellular replication. These results establish for the first time that MNC formation induced by *B. pseudomallei* is not the result of enhanced bacterial replication or survival in host cells, nor is it required to support bacterial growth. The mechanism by which cell fusion is induced by *B. pseudomallei* and enhanced by the absence of AHL synthesis remains for investigation.

Another interesting finding in our study is that neither the *bpsI*12 nor the *bpsI*123 mutants produced detectable AHLs. The lack of AHLs in the *bpsI*12 strain may be due to loss of, or reduced, transcription in stationary phase of *bpsI*3 as result of loss of *bpsI*1, as noted above. Thus the *bpsI*12 mutant may represent a phenotypically triple mutant under these conditions. However, despite this similarity in AHL profile the *bpsI*12 strain did not show the increased MNC phenotype of the triple mutant. We hypothesise that, in cells infected with the *bpsI1*2 mutant, BpsI3 may contribute to production of an AHL that was not detected in our analyses.

Typically, loss of QS results in attenuation of indicators of virulence [23], [24], though an increase in morbidity *in vivo* has also been reported [35]. Previous studies on *B. pseudomallei* have implicated AHL signalling in virulence in the murine model: Mutants in *bps*I1 and *bps*I3 have an increased LD_50_ in hamsters, although the *bps*I1 mutant showed a survival curve similar to wt [25]. Valade et al. [26] showed that a *bps*I1 (“*pm*lI”) mutant had increased LD_50_ in Swiss mice. Conversely, our study found no effect on virulence in mice when all AHL signal production is abolished. Using our in-frame deletion mutants we showed that a variety of AHLs are normally produced but none are detectable in the triple mutant lacking all three synthases. Hence, since the triple mutant showed a major phenotype in cultured cells, but colonization was not significantly reduced as a result of abrogation of AHL production, we conclude that AHL-mediated QS is not necessary for successful acute infection. However, melioidotic lesions, particularly from skin or chronic infections, are noted to contain fused host cells, or granulomas [36]. It thus seems plausible that there remains a role for AHL-mediated QS in chronic infection. Indeed, a recently developed model of murine chronic infection was characterised by MNC-containing lesions [20]. Increased cell fusion in the absence of QS might enhance the formation of such lesions and/or granulomas. The role of AHL synthesis in chronic infection remains to be assessed and the studies reported herein will be useful to test this hypothesis in the setting of chronic disease.

AHL-mediated QS is probably important in survival of *B. pseudomallei* in the environment [26], [29] which might also include resistance to predation by, and/or growth in, amoebae [37]–[40]. In this case, a putative role in chronic infection might be viewed as ‘accidental’ or ‘environmental opportunism’ [16], [37], [41], [42]. It seems likely that QS regulates a gene(s) which is involved in the suppression of MNC formation, identification of which could provide new insight into bacterial mechanisms underlying macrophage cell fusion.
